# Obesity Related Out‐of‐Pocket Expenditure and Productivity Loss: Evidence From Cross‐Sectional Study in Bangladesh

**DOI:** 10.1002/hsr2.72821

**Published:** 2026-07-16

**Authors:** Abdullah Al Marin, Sazia Ahmed, Sajib Chowdhury, Sharmin Akter Keya

**Affiliations:** ^1^ Economics Discipline Khulna University Bangladesh; ^2^ Bangladesh Water Development Board, Government of the People's Republic of Bangladesh, Ministry of Water Resources Dhaka Bangladesh; ^3^ Ahsanullah University of Science and Technology Bangladesh

**Keywords:** balanced diet, body mass index, obesity, physical activity, services

## Abstract

**Background and Aims:**

Obesity has become a major health issue, especially in developing nations. Along with its direct health effects, obesity creates serious financial consequences for individuals, such as out‐of‐pocket expenses as well as productivity loss. Thus, the aim of this cross‐sectional study is to determine the factors influencing productivity loss and out‐of‐pocket expenses associated with obesity.

**Methods:**

The authors analyzed 250 data collected from the southwest region of Bangladesh, applying ordinary least squares and multinomial logistic regression models using STATA.

**Results:**

Descriptive statistics highlight that obese and aged individuals tend to have higher out‐of‐pocket expenditures. Results from the multinomial logit model indicate that factors such as increasing age, higher family income, lack of regular physical activity, frequent fast‐food consumption, and parental BMI significantly influence the likelihood of obesity. In addition, regularity in having lunch or dinner at fast‐food restaurants also has a significant impact on respondents being obese. On the other hand, OLS results reveal that being obese or overweight considerably raises OOP costs and reduces productivity. OOP and productivity loss are further exacerbated by other factors such as family income, eating choices, and annual sick days.

**Conclusions:**

Findings of this study shed importance on promoting healthy eating habits and physical activity as an essential strategy in obesity control. Policymakers should prioritize improved food habits and regular physical exercise as fundamental means of a healthy life.

## 1 Introduction

Obesity, a health and economic phenomenon which has worsened over the last two decades [1], and recognized by the World Health Organization (WHO) as a disease that affects more than 650 million people globally among more than 2 billion overweight individuals [2]. Obesity rate among adults has been predicted to reach over 18% and 21% for men and women, respectively [3]. Besides, obesity related non‐communicable diseases result in more than 5 million deaths yearly, making overweight and obesity the fifth leading cause of death globally [4, 5].

However, the prevalence of increasing obesity, doubling in Southeast Asia, tripling in European regions, has made it a critical global threat for both developed and developing countries [6, 7, 8, 9]. In Bangladesh, obesity is growing alarmingly due to the socioeconomic transitions, rapid urbanizations, and changing lifestyles [10, 11]. Bangladesh's fight against childhood and teenage obesity and overweight issues is becoming increasingly serious [12]. However, previous studies in this context have focused that socioeconomic shifts, processed/fast‐food, heavy diets, sedentary occupations, and changes in lifestyle led people to reduced physical activity, poor dietary patterns, and excessive calorie intake are the root causes behind the rising prevalence of obesity, especially in urban areas [13, 14]. In addition, cultural practices associated with higher BMI and social status reinforce obesogenic behavior among different communities [15]. Consequently, BMI‐related issues have become the primary reasons for the increased likelihood of non‐communicable diseases due to obesity among Bangladeshis, such as diabetes and hypertension [16, 17]. Another study revealed that a negative association exists between self‐perceived nutrition knowledge and BMI [18]. Such trends not only lead us to higher mortality and morbidity risks but also put a burden on the healthcare systems of Bangladesh, underscoring the upsurging economic consequences generated from obesity [19].

While rising obesity and expenditure associated with it have been acknowledged as a clinical and economic threat to Bangladesh, a huge gap exists regarding its household‐level consequences among developing nations. Though several studies have been conducted on high‐income settings and developed nations, but the dual burden due to out‐of‐pocket (OOP) expenditure and costs via productivity losses are yet under researched in countries like Bangladesh. OOP incorporates not only direct medical expenses but also non‐medical expenses such as transportation and informal care [20, 21]. Also productivity losses are measured as lost work hours and forgone earnings caused by that, reduced working capacity [22, 23] and equivalent economic impairments. Understanding the interconnection of obesity‐related factors and the economic consequences via OOP expenditure and productivity losses is important to combat obesity and its economic implications. Therefore, this study aims to investigate the factors of obesity in Bangladesh, focusing on the Khulna region. Parallelly, another key objective is to measure the OOP health expenditures and productivity losses related to obesity. Our research outcomes can contribute to the field of medical science, social science, and health economics, providing helpful regional evidence.

## 2 Materials and Methods

### 2.1 Study Sites and Sampling

Khulna is considered the regional hub in the southwestern region of Bangladesh. It is one of the least populated and progressive districts of Bangladesh with the seventh‐highest literacy rate of around 80% (74.66% national average) and the third largest city of Bangladesh, housing over 2.61 million people (0.71 million in the city area) in approximately 44 km² (Bangladesh Bureau of Statistics, 2022) [24, 25]. In addition, by using national data of Bangladesh, Gupta et al. found that the obesity rate is highest in the urban area of Khulna district (53.7%) [26]. Because of the prevalence of a higher obesity rate, the authors chose Khulna district as the primary study area. Sample distribution is represented in Table 1.

**Table 1:** Sample distribution.

Serial number	Thana name	Sample size
1	Sonadanga	70
2	Daulatpur	65
3	Khan Jahan Ali Thana	60
4	Khalishpur	55
Total		250

*Source:* Authors compilation.

We employ multistage sampling techniques to collect the final data. Khulna metropolitan city comprises nine police station areas. First, we randomly choose four police station areas among the nine of Khulna city as the primary sampling unit. Then, we purposively choose the gyms, yoga centers, swimming clubs, clinics, and hospitals as the secondary sampling units. Finally, we collect the list of participants or patients from the secondary unit and employ systematic random sampling techniques where every 10th respondent is considered for the final survey respondents. If the invited participant refused the final response, we select the immediate next as the final respondent.

We select the respondents based on some selection criteria. The respondents (i) must be over 18 years (ii) must live in urban areas (Khulna city area) for 5 years; (iii) must be listed in the secondary unit list. Based on the secondary survey unit, the author distributed 320 questionnaires; however, 250 respondents were finally selected for the final analysis. Thus, the response rate is 78.10%.

### 2.2 Variable Description

#### 2.2.1 Body Mass Index

Body mass index (BMI) is a traditional approach for measuring body weight [27, 28]. According to the WHO, BMI has been divided into four different bodyweight categories, which are underweight (less than 18.5), healthy/normal weight (18.5–24.9), overweight (25–29.9), and obesity (30 or more) [29]. The condition of being overweight or obese is determined by calculating the BMI, defined as the weight in kilograms divided by height in meters squared [30].

Authors have used self‐reported data supported by the findings of Davies et al., who found a good correlation between self‐reported and direct measures [31]. Data on height were gathered in feet but converted to meters during data entry, whereas data on weight were recorded in kilograms [32].

#### 2.2.2 Physical Exercise, Sleep Cycle, Self‐perceived Nutrition Knowledge, Sedentary Life, and Obesity

Physical inactivity is an influential factor in being overweight and obese. Walking, running, climbing, jumping, and playing are examples of physical activities [33]. It is found that those who have less liking for physical activity are in a positive association with obesity and overweight [34].

Obesity is mainly caused by imbalanced energy intake and expenditure due to a sedentary lifestyle coupled with extra nutrition [35], and people with self‐perceived nutrition knowledge show less prevalence of obesity [36]. Self‐perceived nutrition knowledge, measured as a binary variable by Worsley, is the knowledge about nutrients and nutrition [37]. This has been defined by Azevedo Perry et. al. as “awareness of nutrients and their relevance to health and well‐being, ability to find reliable information about food and/or how foods fit into a balanced diet” in another study [38, 39].

#### 2.2.3 Economic Impact of Obesity

The financial cost of overweight, obese, and morbidly obese individuals with related health issues is substantial. Both direct and indirect economic burdens are related to these medical expenses. Services related to diagnosis, treatment, and prevention are included in direct medical costs. Mortality costs and morbidity costs are related to indirect costs [30]. A study upholds that in 2010, the total cost due to overweight and obesity in Bangladesh was $147.38 million, which is approximately equivalent to 3.69% of the aggregated health care expenditure [19]. Another study demonstrated that out‐of‐pocket expenditure is a very significant part of the economic burden of obesity [40]. The economic impact of obesity is measured via productivity loss and expenditure; out‐of‐pocket expenditure associated with being overweight and obese.

#### 2.2.4 Productivity Loss Expenditure

Productivity loss measures the indirect economic cost resulting from a disease, concentrating on the output cut down by the incapacity of productive human beings to work entirely or at all [41]. It is usually determined using a social perspective, calculating the monetary value of forgone time in both paid and voluntary work [42, 43]. In case of measuring productivity loss, the respondents have been asked about specific cost categories such as costs related to absenteeism, time spent by family members and visitors, and forgone leisure time. The sum of this cost has been used in our analysis. For chronic conditions like obesity, presenteeism is often cited as a significant source of lost productivity, often surpassing losses from absenteeism alone [44].

#### 2.2.5 Out‐of‐Pocket Expenditure

OOP is described as the direct expenditure to health practitioners and providers of health goods and services, paid by households, without the intervention of any third party like insurance or government schemes [45, 46]. Compared to other illnesses, diabetes, cancer, cardiovascular disease, and hypertension cause high out‐of‐pocket expenditure for elderly people [47]. According to Changik, OOP or direct cost estimates for chronic diseases are higher than those for communicable diseases [48]. Therefore, being overweight or obese influences out‐of‐pocket medical expenses. In case of measuring OOP, the respondents have been asked about specific cost categories such as cost of hospitalization special unit (ICU/CCU), nursing home, terminal care, clinic services, physical therapists, nutritionists, nursing services, ambulance, drugs costs, self‐care training services, special diet, diagnostic tests, treatment services, consultation, retraining, transportation and the sum of the costs has been calculated to conduct the analysis of the study.

### 2.3 Statistical Analyses

#### 2.3.1 Regression Models on Obesity

The authors used Microsoft Excel and STATA (version 17) software packages for data analysis. We followed SAMPL guidelines, Lang and Altman, along with another study by Assel et al. for statistical reporting, analysis, and interpretation [49, 50]. Multinomial Logistic Regression is generally effective in situations that classify subjects based on the values of a set of predictor variables [51]. Researchers suggest that the multinomial logistic regression model is appropriate for more than two categorical outcome variables to predict the probabilities of the different possible outcomes of the categorical variable and has been widely used to analyze obesity [52, 53, 54, 55]. The dependent variable, BMI is categorical in nature; therefore, multinomial logistic regression model has been applied for analyzing factors influencing obesity. Side by side, in the light of a study of Wright and Prosser, an ordinary least squares regression (OLS) model on logged expenditures to predict productivity loss and out‐of‐pocket expenditures by BMI class [56, 57, 58]. Equations (1) and (2) exhibit the multinomial logistic regression model where *Y*_*i*_ describes the dependent variable, BMI, which has been divided into three categories (*j *= 3). According to the BMI value, these three categories are normal weight, overweight, and obese. In this study, the base category is the healthy/normal weight, and it remains as the reference class during interpretation. *I*_ij_ refers to the probability value of BMI categories under the dependent (xiβi) variable.

We can calculate the probability as follows:

(1) $$\text{Pr}(\mathrm{yi}=j│\mathrm{xi}=\mathrm{Pij}=\frac{\text{exp}\left(\mathrm{xi}\beta i\right)}{1+\Sigma_{j=3}^{j-1}\text{exp}(\mathrm{xi}\beta j)}$$

For determining the base category,

(2) $$\text{Pr}(y=0│\mathrm{xi}=\mathrm{Pij}=\frac{1}{1+\Sigma_{j=3}^{j-1}\text{exp}(\mathrm{xi}\beta j)}$$

In Equations (1) and (2), x_i_ symbolizes the vector of explanatory variables. *B*_*i*_ represents the coefficient on all those variables. Before applying multinomial regression, variance inflation factor (VIF) and white test have been implemented by turns to check multicollinearity and heteroskedasticity among variables. The outcome from these tests ensures that there is no multicollinearity and heteroskedasticity.

### 2.4 Ethical Consideration

The Khulna University Ethical Clearance Committee of Bangladesh approved this study (Reference No.‐KUECC‐2024‐01‐07). The respondents responded to the questionnaire by being informed of a consent letter, in detail of the research purpose, confidentiality of information, and the right to revoke their engagement prior to justification.

## 3 Results and Discussion

Table 2 summarizes the socio‐economic characteristics and health‐related profiles of respondents. The interviewed individuals are mostly adults and middle‐aged, whose age varies between 36 and 55 years. Regarding parental education, the majority (approximately three‐fourths) of parents hold honors or master's degrees. The sample shows that surveyed individuals belong to high‐ income families with a monthly income exceeding BDT 90,001. Around 63.20% surveyed respondents are male, and most of them are married. Household information reveals nearly 92% of respondents have families with five members, whereas over half of their families are nuclear type, and the majority of the respondents reside with their families. When respondents are asked about their meal preferences at fast‐food restaurants, many of them show a preference to have lunch at fast‐food restaurants. On the other hand, the BMI categorization sample indicates that almost half of the respondents are overweight, 28.40% are obese, and the remaining have a normal weight. Lastly, data on yearly out‐of‐pocket expenditure shows two‐fifths of the sample pay BDT 12,001–15,000 as out‐of‐pocket expenditure in a year.

**Table 2:** Summary statistics.

Variable name	Observation	Percent	[95% Conf. interval]	
Age category				
Less than 35	49	19.60	0.15	0.25
36–45	81	32.40	0.26	0.38
46–55	64	25.60	0.20	0.31
More than 55	56	22.40	0.17	0.28
Father education				
Secondary complete	37	14.80	0.10	0.19
Higher secondary complete	25	10.00	0.06	0.14
Honors and masters	188	75.20	0.69	0.80
Mother education				
Secondary complete	18	7.20	0.04	0.11
Higher secondary complete	56	22.40	0.17	0.28
Honors and masters	176	70.40	0.64	0.75
Income category				
Less than 50,000	58	23.20	0.18	0.28
50,001–70,000	52	20.80	0.16	0.26
70,001–90,000	31	12.40	0.08	0.17
More than 90,001	109	43.60	0.37	0.49
Gender				
Female	92	36.80	0.31	0.43
Male	158	63.20	0.57	0.69
Marital status				
Unmarried	17	6.80	0.04	0.10
Married	233	93.20	0.89	0.95
Family status				
Joint	111	44.40	0.38	0.50
Nuclear	139	55.60	0.49	0.61
Household size				
5	229	91.60	0.87	0.94
6	9	3.60	0.01	0.06
7	9	3.60	0.01	0.06
8	3	1.20	0.00	0.03
Meal prefers to consume at a fast‐food restaurant				
Breakfast	83	33.20	0.27	0.39
Lunch	88	35.20	0.29	0.41
Dinner	79	31.60	0.26	0.37
Current living arrangement				
With family	227	90.80	0.86	0.93
Mess	23	9.20	0.06	0.13
Body mass index				
Normal weight	57	22.80	0.18	0.28
Overweight	122	48.80	0.42	0.55
Obesity	71	28.40	0.23	0.34
Out‐of‐pocket expenditure category				
Less than 12,000	92	36.80	0.31	0.43
12,001–15,000	101	40.40	0.34	0.46
More than 15,000	57	22.80	0.18	0.28

### 3.1 Out‐of‐Pocket Expenditure Condition Among BMI and Age Group

**Figure 1:**
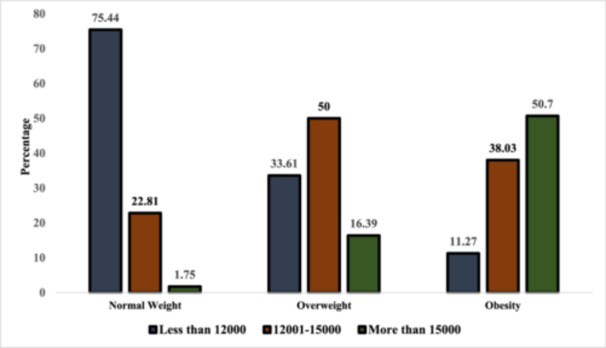
Out‐of‐Pocket Expenditure across different BMI Group. *Source:* Authors Compilation.

Figure 1 illustrates the frequency of out‐of‐pocket expenditure of respondents across different BMI groups. The data reveal that OOP is lower in the normal weight group and higher in the obesity group. Almost half of the overweight individuals spend BDT 12,001–15,000 as OOP per year. Notably, the figure highlights that obese individuals may experience high (more than 15,000) OOP compared to the other two BMI categories.

**Figure 2:**
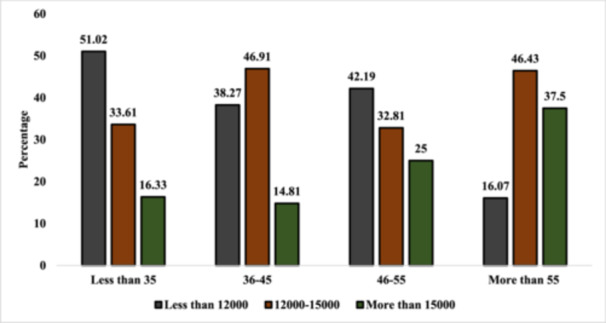
Out‐of‐pocket expenditure across different age groups. *Source:* Authors' compilation.

Figure 2 presents the distribution of out‐of‐pocket expenditure across different age groups. The result shows that most individuals (34%–46%) spend yearly BDT 12,000–15,000 as indirect healthcare cost. Surprisingly, as people age, they tend to have higher out‐of‐pocket expenditure than other age groups. Previous literature mentioned that, with age due to changes in metabolism, physical activity, and dietary patterns, people get obese or overweight and have to spend more indirect healthcare costs [59].

### 3.2 Chronic Disease Among BMI Group

Table 3 illustrates the prevalence of chronic diseases across different BMI groups. Among the five types of chronic diseases, Type II Diabetes is the most common across all BMI categories, with overweight individuals being particularly affected. Both normal‐weight and obese individuals also show a high incidence of Type II Diabetes. Additionally, some overweight and obese individuals experience hypertension. Furthermore, a small number of overweight and obese respondents suffer from heart and liver diseases.

**Table 3:** Chronic disease across different BMI group.

Diseases	Normal weight *N* (%)	Overweight *N* (%)	Obesity *N* (%)
Dyslipidemia	0 (0.00)	5 (4.10)	5 (7.04)
Heart disease	2 (3.51)	6 (4.92)	9 (12.68)
Hypertension	6 (10.53)	18 (14.75)	14 (19.72)
Liver disease	5 (8.77)	9 (7.38)	2 (2.82)
Type II diabetes	44 (77.19)	84 (68.85)	41 (57.75)
Total	57 (100.00)	122 (100.00)	71 (100.00)

### 3.3 Factors of Obesity

Table 4 reports the results of a multinomial logistic regression analysis that examines the factors influencing the likelihood of being overweight or obese compared to having a normal weight. In summary, the table shows that mothers' education, family income, meal choices, living arrangements, fathers' BMI, age, gender, and the frequency of fast‐food intake are important predictors of being overweight and obese.

**Table 4:** Factors of obesity.

	Overweight		Obesity	
Variable name	RRR (std. err.)	*p* value	RRR (std. err.)	*p* value
Age	1.07 (0.04)	0.06	1.14 (0.04)	0.00
Gender	1.41 (0.76)	0.52	5.76 (3.84)	0.00
Fathers' education	1.10 (0.11)	0.32	1.03 (0.13)	0.80
Mothers' education	1.54 (0.25)	0.01	2.12 (0.40)	0.00
Log of family income	8.77 (14.93)	0.20	95.89 (201.95)	0.03
Number of times consuming fast food	1.97 (0.52)	0.01	3.42 (1.03)	0.00
Meal prefers to consume at a fast‐food restaurant (base: breakfast)				
Lunch	55.72 (56.51)	0.00	9.14 (11.10)	0.06
Dinner	20.67 (14.44)	0.00	17.03 (13.57)	0.00
Current living arrangement	16.36 (27.35)	0.09	227.97 (406.28)	0.00
Father's BMI Index	1.42 (0.17)	0.00	1.68 (0.22)	0.00
Suffering from any chronic disease	0.369 (0.37)	0.33	0.32 (0.36)	0.31
Performing exercise	0.126 (0.11)	0.02	0.12 (0.11)	0.02
Yearly number of sickness day	0.5 (0.21)	0.10	0.49 (0.23)	0.13
Self‐perceived nutrition knowledge	0.93 (0.65)	0.92	2.67 (2.08)	0.20
Constant	−30.58 (11.32)	0.00	58.39 (13.71)	0.00
Observation	250		250	

*Note:* N.B.: base: normal weight; RRR indicates relative risk ratio; std. err. indicates standard errors; CI indicates confidence interval.

The regression results in Table 4 show a positive relation between age and the risk of getting overweight or obese, which is in line with the previous findings [60, 61, 62]. Increasing age raises the likelihood of being obese or overweight by 14% (*p* < 0.10) and 7% (*p* < 0.00) [62]. Interestingly, the study shows that males are significantly more likely than females to be obese (*p* < 0.00), even though gender does not significantly affect obesity status. Numerous studies have reported this gender difference in obesity risk [59, 63].

Furthermore, there is a correlation between higher mothers' education and a child's likelihood of being overweight or obese (*p* < 0.05, 0.00). This finding contrasts with some previous research that suggests better health habits and knowledge are generally associated with healthier weight outcomes in children who have higher parental education, especially maternal education [64]. However, the study's discovered positive impact may be because of complex socioeconomic dynamics, as higher maternal education is related to more resources that could indirectly encourage weight gain‐related habits like eating more calorie‐dense foods or engaging in less physical activity [61]. The results are aligned with previous findings and conclude that there is a high probability of obesity in high‐income families [60, 61, 65, 66]. A complex relationship between wealth and obesity suggests that increased financial solvency may increase access to calorie‐rich foods and lead to more lavish lifestyles, which in turn raise obesity rates.

Frequent consumption of rich foods, such as whole grains, meat, and fish, increases the probability of being obese and overweight [67]. This study supports this finding with a statistically significant positive relation between fast food intake and risk of getting obese or overweight. Relative risk ratio indicates that with one additional frequency of fast‐food intake increases the risk of being overweight by 97% (*p* < 0.05, 0.00). Meal preferences at fast‐food restaurants, an important predictor variable shows statistically significant positive relationship with being obese and overweight (*p* < 0.00, 0.05, 0.10). Specifically, the result indicates that individuals who prefer lunch over breakfast at fast‐food establishments are more likely to be overweight and obese. Supporting the findings of Jakubowicz et al. [68], this study also manifests that foods that are usually eaten for lunch and dinner may put you at risk for weight gain because they are often higher in calories and lower in nutrients than breakfast foods. Moreover, late‐night eating is related to abnormalities, which impair metabolism and increase obesity [69]. On the other hand, living in a social and environmental setting is a significant indicator in the development of obesity as well as overweight conditions. According to Yen et al., living arrangements have impact on eating practices, levels of physical activity, food hygiene, and general lifestyle choices of individuals [70].

Children are more likely to become obese if there is a family history of obesity, according to Hwang and Bang and Gokosmanoglu et al. [61, 67]. Our study also shows that parental BMI is a key factor contributing to being overweight and obesity, which is in line with other findings. According to Reilly et al., paternal BMI is probably a reflection of both genetic predispositions that can make offspring more likely to have a higher body mass index (BMI) [71]. In line with a few previous findings this study also concludes that regular exercise reduces the risk of becoming overweight or obese, as the relative risk ratios (RRRs) for both overweight and obesity are statistically significant (*p* < 0.00) [61, 65, 66].

### 3.4 Factors of Out‐of‐Pocket Expenditure and Productivity Loss

Table 5 shows the factors influencing respondents' out‐of‐pocket expenditure and productivity losses. The findings show that being obese or overweight considerably raises OOP costs and reduces productivity. OOP and productivity loss are further exacerbated by other factors such as family finances, eating choices, and annual sick days.

**Table 5:** Factors of OOP and productivity loss.

Variable Name	OOP		Productivity loss	
	Coefficient	*p* value	Coefficient	*p* value
Age	0.0003 (0.0003)	0.11	0.0008 (0.0005)	0.15
Gender	−0.0009 (0.008)	0.30	−0.009 (0.009)	0.12
Fathers education	0.002 (0.001)	0.16	0.002 (0.002)	0.11
Mothers education	−0.0006 (0.001)	0.18	−0.0003 (0.002)	0.11
Log of family income	−0.024 (0.017)	0.13	0.079 (0.046)	0.09
BMI category (base: normal weight)				
Overweight	0.065 (0.012)	0.00	0.034 (0.017)	0.04
Obesity	0.113 (0.015)	0.00	0.131 (0.019)	0.00
Number of times consuming fast food	0.002 (0.003)	0.11	−0.002 (0.006)	0.16
Meal prefers to consume at a fast‐food restaurant (base: breakfast)				
Lunch	0.027 (0.010)	0.00	−0.001 (0.013)	0.29
Dinner	−0.012 (0.012)	0.11	0.033 (0.021)	0.16
Current living arrangement	−0.013 (0.017)	0.14	−0.033 (0.011)	0.10
Father's BMI index	0.002(0.002)	0.05	0.002(0.001)	0.11
Suffering from any chronic disease	−0.009 (0.010)	0.12	−0.004 (0.017)	0.14
Performing exercise	0.009 (0.009)	0.11	0.0104 (0.026)	0.13
Yearly number of sick days	0.006(0.004)	0.09	−0.007 (0.006)	017
Self‐perceived nutrition knowledge	−0.017 (0.009)	0.08	−0.016 (0.019)	0.12
Constant	4.060(0.091)	0.00	4.179 (0.191)	0.00
Observations	250		250	
R‐squared	0.413		0.376	

*Note:* Robust standard errors in parentheses.

The OLS result in Table 5 indicates a strong correlation between obesity, productivity loss, and family income. With 1% increment in family income results in a 7.9% rise in productivity loss, which is statistically significant at the 10% level. This finding exhibits that high‐income jobs generally come with more time demand and stress. However, authors don't find any significant impact of family income on out‐of‐pocket expenses, which is beyond our expectations. On the other hand, earlier studies like Okello and Njeru found a correlation between higher household income and higher out‐of‐pocket spending [72]. There could be variations in the research populations, the economic environments, or the variables examined that account for the disparity between these studies.

Additionally, the study demonstrates that overweight or obese people pay much more out‐of‐pocket than normal‐weight individuals. This result is in line with previous studies that strengthen the financial burden of obesity on individuals, particularly healthcare expenditure [73]. However, compared to normal weight, overweight or obese individuals lose more productivity, which is statistically significant at 5% and 1% levels, respectively. These findings emphasize double economic costs of obesity; one is direct healthcare cost, and another is indirect costs from missed. Regression results indicate fathers' BMI is positively associated with respondents' out‐of‐pocket expenses. A one‐unit increase in the father's BMI leads to a 0.2 unit increase in out‐of‐pocket expenditure, suggesting that family health history and parental weight status can have long‐term implications for OOP. This result is also in line with earlier studies that showed health behaviors and related expenses are passed down across generations [74].

Furthermore, out‐of‐pocket costs at fast‐food restaurants are influenced by meal preferences; those who choose lunch over breakfast will pay more, which is significant at the 1% level, due to variations in the nutritional value, portion sizes, or meal composition of fast‐food restaurants' breakfast and lunch options. Side by side, this study discovers a positive association between the number of annual sick days and out‐of‐pocket expenses. The coefficient of Table 5 points that, with each additional sick day, OOP is expected to rise by 0.6 units. Finally, authors found that individuals with more knowledge about health and nutrition tend to experience lower OOP, which supports the findings of previous studies like Cawley and Meyerhoefer, who stress the importance of health education in forming individuals' eating behavior and lessening healthcare expenses [75]. This result indicates that healthy food choices, handling health issues consciously, taking preventive care, and growing healthy food habits can lower the financial costs of ill health.

Despite some valuable insights resulting from this study, it has several limitations, like the primary focus of this research is on obese and health‐conscious people; however, the findings are not generalized for all people. Besides, this study uses self‐reported height and weight data, which probably creates systematic bias. Wide standard errors for some coefficients indicate possible model instability related to sample size and model complexity. Furthermore, the self‐reported measurement of nutrition knowledge may have introduced reporting bias.

## 4 Conclusion

Obesity is considered a serious health crisis that endangers the healthy lifestyle of people. Recently in Bangladesh, the obesity rate among people of different ages is increasing at an alarming rate. So, this study aims to investigate the factors influencing obesity among people and how obesity influences out‐of‐pocket and productivity loss expenses. Through considering respondents' socio‐demographic and health profile, this study found variables like mother's education, family income, meal choices, living arrangements, fathers' BMI, age, and fast‐food consuming frequency as important predictors of overweight and obesity. On the other hand, authors also found a significant association among increasing out‐of‐pocket expenditure, productivity loss, and participants' level of BMI. Obese and overweight individuals usually experience more OOP and productivity loss compared to normal people. Obese individuals may face serious health complications, including cardiovascular disease, diabetes, cancer, etc. Therefore, doing regular physical exercise, maintaining a balanced diet plan, and an appropriate sleep cycle are required to avoid and mitigate the challenges of obesity and its economic burden on people. The results of this study suggest a need to further strengthen awareness‐raising activities aimed at preventing obesity.

## Author Contributions

**Abdullah Al Marin:** conceptualization, investigation, writing – original draft, methodology, formal analysis. **Sazia Ahmed:** supervision, conceptualization, investigation, visualization, methodology. **Sajib Chowdhury:** conceptualization, methodology, validation, writing – review and editing, software. **Sharmin Akter Keya:** conceptualization, investigation, writing – review and editing, visualization, validation.

## Funding

The authors have nothing to report.

## Conflicts of Interest

The authors declare no conflicts of interest.

## Transparency Statement

The corresponding author, Sajib Chowdhury, affirms that this manuscript is an honest, accurate, and transparent account of the study being reported; that no important aspects of the study have been omitted; and that any discrepancies from the study as planned (and, if relevant, registered) have been explained.

## Data Availability

The data that support the findings of this study are available from the corresponding author upon reasonable request.
